# Impact of the number of prior chemotherapy regimens on outcomes for patients with metastatic breast cancer treated with eribulin: A post hoc pooled analysis

**DOI:** 10.1111/tbj.13686

**Published:** 2019-11-29

**Authors:** Javier Cortes, Chris Twelves

**Affiliations:** ^1^ IOB Institute of Oncology Quironsalud Group Madrid & Barcelona Spain; ^2^ Vall d´Hebron Institute of Oncology Barcelona Spain; ^3^ Leeds Institute of Medical Research at St James's University of Leeds and Leeds Teaching Hospitals Trust Leeds UK

**Keywords:** advanced breast cancer, efficacy analysis, eribulin, overall survival, safety

## Abstract

In a pivotal phase 3 study (Study 305), eribulin mesylate improved overall survival (OS) in patients with previously treated metastatic breast cancer (MBC) compared with treatment of physician's choice (TPC). This post hoc, pooled subgroup analysis of two phase 3 studies (Study 305 and Study 301) reports the influence of the number of prior chemotherapy regimens (0‐6) on OS in patients with locally advanced/MBC randomized to eribulin versus TPC/capecitabine. Patients with ≤ 3 prior chemotherapies for locally advanced/MBC had longer median OS with eribulin (15.3 months) versus control (13.2 months; hazard ratio, 0.858; *P* = .01).

## 1 BACKGROUND

Metastatic breast cancer (MBC) remains incurable, and few cytotoxic agents prolong overall survival (OS). Several cytotoxic therapies are approved for treating patients with MBC, and current clinical guidelines generally recommend sequential monotherapies, but not a preferred sequence of administration.^1^

Eribulin, a synthetic analogue of halichondrin B, inhibits microtubule growth, blocks cell‐cycle progression, and induces apoptosis of tumor cells.^2^ In preclinical studies, eribulin induced vascular remodeling and increased tumor perfusion^3^; similarly, noncytotoxic effects have been demonstrated clinically.^4^ Eribulin mesylate (eribulin) is approved in the United States for the treatment of patients with MBC after ≥2 prior chemotherapies for metastatic disease; additionally, it is approved in the European Union for locally advanced/MBC patients with ≥1 prior chemotherapies for advanced disease. Prior treatments should include a taxane and an anthracycline.

Two randomized, open‐label, phase 3 trials (Study 305/EMBRACE and Study 301 [ClinicalTrials.gov: NCT00388726 and NCT00337103, respectively]) assessed the efficacy and safety of eribulin in pretreated patients with locally recurrent/MBC.^5^, ^6^ In a previous pooled analysis of these 2 studies,^7^ median OS was 15.2 months (eribulin) versus 12.8 months (control arm: hazard ratio [HR], 0.85; 95% confidence interval [CI] 0.77‐0.95; *P* = .003); OS favored eribulin in all analyzed subgroups including human epidermal growth factor receptor 2 (HER2)‐negative disease (HR, 0.82; *P* = .002), and triple‐negative disease (HR, 0.74; *P* = .006). These findings were supported by another pooled analysis in patients with ≥1 prior chemotherapy regimens.^8^ Here, we report an exploratory, post hoc, pooled subgroup analysis of the influence of the number of prior chemotherapy regimens on OS using data from EMBRACE and Study 301.

## 2 METHODS

Both trials enrolled women aged ≥18 years, with previously treated locally recurrent/MBC.^5^, ^6^ OS was compared between eribulin and treatment of physician's choice [TPC] (EMBRACE) or capecitabine (Study 301) in the intent‐to‐treat (ITT) populations.^5^, ^6^ HRs for EMBRACE only were based on a Cox regression model including HER2/neu status, geographical region, and prior capecitabine treatment as stratification variables.^5^ HRs for pooled data were estimated based on the Cox model with stratification factors (HER2 status, region, prior capecitabine use, and study). Median OS was adjusted by study (defined in Twelves et al, 2014^7^) and *P*‐values were estimated by stratified log‐rank test. An exploratory comparative analysis of OS grouped by ≤3 versus >3 prior treatments, and by individual number of prior lines of treatment (ie, 0, 1, 2, 3, 4, 5, and 6), for locally advanced/MBC was completed using data pooled from both studies except as noted (ie, data on ≥5 prior lines of therapy are from EMBRACE only). A pooled analysis of safety data was not possible because the studies used different versions of the Medical Dictionary for Regulatory Activities (version 10.0 for EMBRACE, version 14.1 for Study 301).

## 3 RESULTS

### 3.1 Patients

In EMBRACE, patients were randomized 2:1 to receive eribulin (1.4 mg/m^2^ [equivalent to 1.23 mg/m^2^ when expressed as a free base] intravenously on days 1 and 8 every 21 days; n = 508) or TPC (n = 254).^5^ In Study 301, 554 patients were randomized to receive eribulin and 548 to receive capecitabine.^6^ Patient characteristics have been previously reported.^5^, ^6^ Almost all (99%) patients had received prior anthracycline and taxane therapy.^5^, ^6^ In EMBRACE, the median number of prior chemotherapy regimens for locally advanced/MBC was 3 (with approximately one‐quarter having >3 and three‐quarters having ≤3). In Study 301, only 1 patient (a protocol deviation) received >3 prior chemotherapy regimens for locally advanced/MBC.

### 3.2 Post hoc efficacy analysis

**Figure 1:**
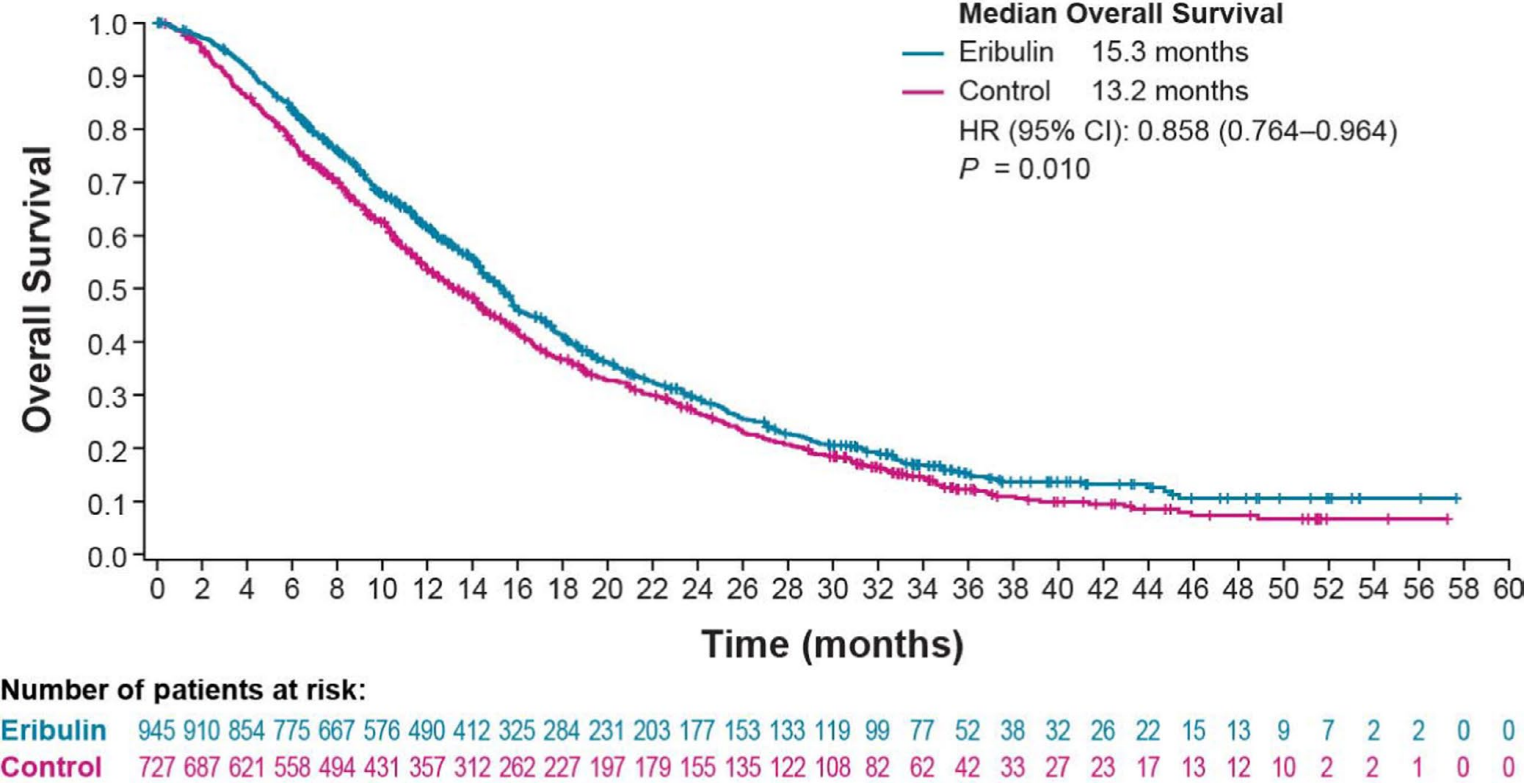
Overall Survival Curves for Patients Pooled From Study 301^6^ and EMBRACE^5^ (ITT Population). Populations comprised those who received ≤3 prior chemotherapy regimens for advanced or metastatic breast cancer. Note, CI, confidence interval; “control,” control treatments were either treatment of physician's choice or capecitabine; HER2, human epidermal growth factor receptor 2; HR, hazard ratio; ITT, intent‐to‐treat population. Overall survival and medians were calculated per study adjustment following the same method outlined previously.^7^ *P*‐value was based on stratified log‐rank test. The HR (eribulin/control) and the corresponding 95% CIs were generated based on the Cox regression model, with stratification factors of study, region (North America/Western Europe/Australia, Latin America/South Africa, Eastern Europe, and Asia), prior capecitabine use, and HER2/neu status

This subgroup analysis demonstrated a nominally significant difference in median OS with eribulin treatment (ITT group, n = 945) versus control (n = 727) in patients who received ≤ 3 prior chemotherapy regimens for locally advanced/MBC (15.3 vs 13.2 months, respectively; HR, 0.858; *P* = .01; Table 1, Figure 1). In EMBRACE, patients with >3 prior regimens for locally advanced/MBC had a median OS in the eribulin (n = 117) versus TPC (n = 73) ITT groups of 11.7 versus 10.0 months, respectively; this improvement was again nominally significantly different in patients with ≤3 prior chemotherapy regimens for locally advanced/MBC (eribulin, n = 391; TPC, n = 180; 13.3 vs 10.7 months, respectively; *P* = .039; Table 1).

**Table 1:** Overall survival for locally advanced/MBC patients with ≤3 or >3 prior chemotherapy regimens

Parameter	Patients randomized to receive		Median survival difference
	Eribulin	Control	
≤3 Prior chemotherapy regimens (EMBRACE)			
n	391	180	2.6 mo^a^ 78 d
Median overall survival	13.3 mo^a^ 404 d	10.7 mo^a^ 326 d	
95% CI, days	365.0‐454.0	282.0‐380.0	
*P*‐value^b^	0.039		
Hazard ratio^c^ (eribulin vs TPC)	0.774		
95% CI	0.606‐0.988		
≤3 Prior chemotherapy regimens (pooled data from Study 301 and EMBRACE)			
n	945	727	2.1 mo 64 d
Median overall survival	15.3 mo 466 d	13.2 mo 402 d	
95% CI, days	438.3‐484.0	365.3‐441.3	
*P*‐value^b^	0.010		
Hazard ratio^d^ (eribulin vs control^e^)	0.858		
95% CI	0.764‐0.964		
>3 Prior chemotherapy regimens (EMBRACE)			
n	117	73	1.7 mo^a^ 51 d
Median overall survival	11.7 mo^a^	10.0 mo^a^	
	355 d	304 d	
95% CI, days	281.0‐420.0	191.0‐547.0	
*P*‐value^b^	0.607		
Hazard ratio^c^ (eribulin vs TPC)	0.899		
95% CI	0.600‐1.348		

Note

CI, confidence interval; HER2, human epidermal growth factor receptor 2; ITT, intent‐to‐treat; TPC, treatment of physician's choice.

a A conversion factor of 30.4375 was used to convert number of days into months.

b Based on stratified log‐rank test, for Study 301, strata included HER2/neu status (clinical database) and geographical region; for analyses of EMBRACE, strata included HER2/neu status (clinical database), geographical region, and prior capecitabine treatment; for pooled analyses, strata included study, geographical region, prior capecitabine use, and HER2/neu status.

c Hazard ratios and the corresponding 95% CI were generated based on a Cox regression model with stratification factors of: HER2/neu status, prior capecitabine treatment (for EMBRACE), and geographical region.

d Hazard ratios and the corresponding 95% CI were generated based on the Cox regression model, with stratification factors of: study, geographical region (North America/Western Europe/Australia, Latin America/South Africa, Eastern Europe, Asia), prior capecitabine use, and HER2/neu status.

e The control treatments were TPC for EMBRACE and capecitabine for Study 301.

Additional exploratory pooled post hoc analysis for patients receiving 0‐6 prior lines of therapy showed a trend for higher OS in patients treated with eribulin compared with control (Table 2), and this trend was emphasized in those patients with 0‐3 prior lines of therapy compared with those who had been more heavily pretreated. However, caution is warranted due to the low number of patients, especially in the latter subgroups.

**Table 2:** Overall survival by number of prior chemotherapy regimens for locally advanced or metastatic breast cancer

Number of prior regimens	Overall survival, months (95% CI)		Hazard ratio^b^ (95% CI)	*P*‐value^c^, ^d^
	Patients randomized to receive			
	Eribulin	Control^a^		
0	n = 117	n = 105	n = 222	.5537
	15.57 (13.11‐18.79)	14.39 (11.96‐19.02)	0.908 (0.66‐1.249)	
1	n = 288	n = 300	n = 588	.0723
	15.8 (14.82‐18.10)	14.19 (11.96‐16.07)	0.846 (0.705‐1.016)	
2	n = 373	n = 236	n = 609	.4757
	14.85 (12.45‐16.00)	13.31 (11.73‐15.61)	0.927 (0.754‐1.14)	
3	n = 167	n = 87	n = 254	.0098
	14.26 (11.99‐15.34)	9.23 (6.70‐11.99)	0.608 (0.416‐0.887)	
4	n = 92	n = 56	n = 148	.9510
	12.98 (9.46‐15.28)	13.14 (7.06‐18.6)	0.984 (0.608‐1.592)	
5	n = 21	n = 13	n = 34	.6643
	8.87 (4.57‐13.11)	5.62 (3.61‐NE)	1.265 (0.436‐3.671)	
6	n = 4	n = 5	n = 9	.1098
	8.9 (NE‐NE)	3.65 (NE‐8.71)	0.185 (0.019‐1.838)	

Note

CI, confidence interval, HER2, human epidermal growth factor receptor 2; ITT, intent‐to‐treat; NE, not evaluable; TPC, treatment of physician's choice.

a The control treatments were TPC for EMBRACE and capecitabine for Study 301.

b Hazard ratios and the corresponding 95% CI were generated based on the Cox regression model, with stratification factors of: study, geographical region (North America/Western Europe/Australia, Latin America/South Africa, Eastern Europe, Asia), prior capecitabine use, and HER2/neu status.

c Based on stratified log‐rank test, for pooled analyses (number of prior line[s] of therapy: 0‐4), strata include study, geographical region, prior capecitabine use, and HER2/neu status.

d Based on stratified log‐rank test, for analyses of EMBRACE (number of prior lines of therapy: 5‐6), strata include HER2/neu status (clinical database), geographical region, and prior capecitabine treatment.

### 3.3 Safety

The number of prior chemotherapies appeared not to affect the safety of eribulin in EMBRACE. Although neutropenia and asthenia/fatigue rates were higher with eribulin treatment compared with control, the incidences of both were similar regardless of whether patients had ≤3 or >3 prior regimens (neutropenia, 51.7% for both subgroups; asthenia/fatigue, 53.2% vs 55.1% for ≤3 vs >3, respectively). For patients in the TPC group, the incidences of neutropenia (30.9% vs 25%) and asthenia/fatigue (40.4% vs 36.8%) were numerically higher in patients having ≤3 prior regimens compared with those having >3 prior regimens.

## 4 DISCUSSION

This exploratory subgroup analysis of EMBRACE^5^ and Study 301^6^ shows that the OS benefit conferred by eribulin over TPC/capecitabine is predominantly seen in patients who had fewer prior regimens (≤3) for locally advanced/MBC with a median OS benefit of 2.1 months. This difference in OS was also observed in EMBRACE alone (≤3 prior regimens, 2.6 months; >3 prior regimens, 1.7 months); the number of prior regimens appeared not to affect the safety of eribulin.

The pooled subgroup analysis by number of prior regimens showed that eribulin conferred an OS benefit of 1.2, 1.6, and 1.5 months for patients treated with 0, 1, or 2 prior regimens for locally advanced/MBC, respectively, with a 5.0‐month OS benefit observed for patients with 3 prior regimens (HR, 0.608; *P* = .0098). Patient numbers were, however, not large enough to draw conclusions regarding the relative efficacy of eribulin in patients who had received 0, 1, 2, or 3 prior chemotherapy regimens. Benefit from eribulin appeared reduced in more heavily pretreated patients, but patient numbers were small, especially for those with 6 prior regimens (9 patients).

The greater benefits of eribulin when used in earlier‐line settings are supported by other studies.^9^, ^10^ In a post hoc subgroup analysis of patients (n = 392) in Study 301, treated in the second‐line setting,^10^ median OS was longer in those with HER2‐negative MBC receiving eribulin versus capecitabine (16.1 vs 13.5 months, respectively; HR, 0.77; *P* = .026). A large‐scale clinical study in patients with advanced/MBC, randomized to receive eribulin or vinorelbine,^9^ also achieved its primary end point of prolonged progression‐free survival (HR, 0.80; *P* = .036). Again, the benefit in progression‐free survival from eribulin was seen in patients who had received fewer prior regimens for metastatic disease (≤2; HR, 0.69; 95% CI 0.53‐0.91) but not in those who had been more heavily pretreated (>2; HR, 0.91; 95% CI 0.66‐1.25).^9^

Despite the post hoc nature and small sample size (especially for patients with >3 prior regimens for locally advanced/MBC), this study suggests there may be potential benefit in using eribulin to treat patients with locally advanced/MBC sooner rather than later. As there is considerable attrition in patients receiving successive lines of therapy, it is appropriate that treatments demonstrating the greatest benefit are used earlier for patients with locally advanced/MBC.

## 5 CONCLUSIONS

Patients who have received 3 or fewer regimens for locally advanced/MBC showed an improvement in OS if treated with eribulin rather than with TPC/capecitabine. Clinicians should consider the use of eribulin as indicated and available for the treatment of such patients.
